# Negative Impact of Dental Wastewater on the Environment and Human Health: A Scoping Review

**DOI:** 10.1002/wer.70243

**Published:** 2025-12-18

**Authors:** Giordana Picolo Furini, Rafaela Munz Belarmino, Lilian Rigo

**Affiliations:** ^1^ ATITUS Education Passo Fundo RS Brazil

**Keywords:** dental materials, dental waste, environmental pollution, wastewater

## Abstract

This study aimed to analyze evidence on the physical, chemical, and microbiological risks associated with dental wastewater (DWW) and its impact on the environment and human health. As part of a scoping review, we searched the PubMed, Scopus, and Web of Science databases for studies that described DWW management, characterization, filtration, and associated risks. The search was limited to studies published in English, including experimental, laboratory, observational studies, and reviews. We extracted the study design, country of origin, sample location, components, objectives, and results. Using VOSviewer software, an analysis of author coauthorship and keyword co‐occurrence was conducted. Environmental and human risks were examined, and strategies to minimize damage were discussed. The search initially yielded 1967 articles until June 2024. After removing duplicates and applying exclusion criteria, 29 articles were selected for inclusion. Most studies (55.1%) were experimental, with heavy metals being the most frequently studied pollutants (60%), particularly mercury (Hg). Microbiological analyses appeared in six studies (20.6%), and bisphenol A in two studies (6.9%). The environmental pollutant potential of DWW was reported in 22 studies (75.8%), while only five studies (17.2%) documented risks to humans. In conclusion, DWW poses significant environmental hazards due to its toxic composition and pollutant potential. Although evidence on human health risks is still limited and fragmented, preliminary findings suggest possible concerns that warrant attention. These results highlight the urgent need for more comprehensive studies and support the implementation of regulatory and management strategies to mitigate environmental and potential human health impacts.

## Summary

- Dental wastewater (DWW) contains toxic components, including heavy metals, bisphenol A, and microorganisms.
- Mercury was the most frequently studied pollutant, found in 60% of included studies.
- Environmental risks of DWW were identified in over 75% of the studies, while human health risks were less frequently reported.
- Microbiological contamination and antimicrobial resistance were addressed in a minority of studies.
- There is an urgent need for regulatory actions and filtration strategies to mitigate DWW‐related hazards.

## Graphical abstract

Dental wastewater (DWW) may carry hazardous contaminants (toxic metals, chemicals, and microorganisms) from clinical procedures into municipal sewers and wastewater treatment plants, with potential release to receiving waters. Because treatment may not fully remove all pollutants, the most effective prevention point is source control. Amalgam separators, chair‐side filters, pretreatment, and best practices reduce pollutant loads before discharge, helping protect ecosystems and limit indirect human exposure.

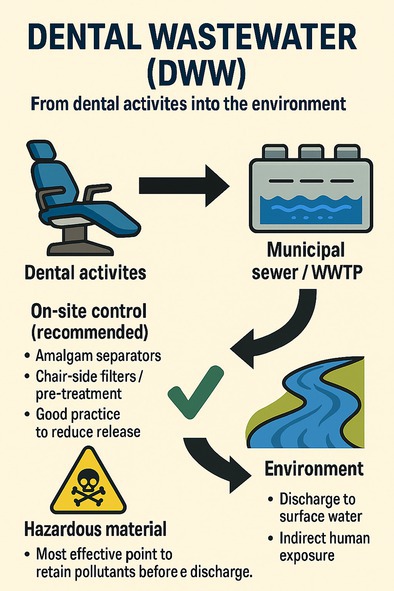

## 1 Introduction

Dental wastewater (DWW) comprises liquid waste from dental equipment, directed to the domestic sewage system. Therefore, DWW is classified as wastewater from human and domestic activities, suitable for disposal into the urban sewage system (Cataldi et al. 2017). These wastes may contain hazardous substances such as pharmaceutical residues, chemicals with their respective toxicities, pathogens, radioisotopes, and a significant concentration of mercury (Hg). (Rani et al. 2015). Due to the presence of these substances, DWW can pose a chemical, biological, and physical risk to public health and the environment (Mulligan et al. 2021; Shraim et al. 2011).

To provide context, dental chair units use water to cool and irrigate their instruments, tooth surfaces, and to provide rinsing water during dental treatment; in addition, it is also supplied to the filling cup outlet of the dental units, which is used by patients for mouth rinsing, and to the rinsing bowl outlet that rinses the cuspidor of the dental units (O'Donnell et al. 2011). Each dental procedure contributes to the composition of the DWW. The cuspidor and vacuum suction system, for example, collect not only saliva and blood, but also a significant volume of irrigation water from the triple syringe and handpieces (high‐ and low‐speed handpieces) (Cataldi et al. 2017).

Dental practice is a globally ubiquitous and large‐scale source of effluents. To contextualize the sector, the United States, the European Union, and Brazil alone account for over one million active dentists combined, approximately 202,485 in the United States (ADA, HPI 2024), 363,162 in the European Union (Eurostat 2023), and 450,000 in Brazil (CFO 2025). The cumulative impact of this healthcare network is substantial, not only in the number of establishments but also in the volume of waste generated. Design regulations for dental facilities estimate that each chair can discharge a DWW volume in the range of 189–757 L (50–200 gal) per day (SORA 2017). This significant effluent volume is identified as one of the main sources of heavy metal pollution in municipal sewage. In fact, literature reviews estimate that dental offices are responsible for a significant fraction of the total Hg load reaching wastewater treatment plants (WWTPs), with some reports indicating contributions ranging from 8% to 14% (Fan et al. 2002) and potentially reaching up to 70% of the total daily Hg load in some estimates (Jamil et al. 2016).

In this scenario, dental materials that stand out in research literature and have limited regulatory status in a few countries are Hg residues from amalgam restorations and radiographic film processing. However, contemporary dental materials go far beyond these. Currently, the most widely used dental material for restorative treatments and substitutes for amalgam restorations is resin‐based (Hg‐free), which may have a concurrent effect on DWW (Binner et al. 2022; Mulligan et al. 2021).

In recent decades, the release of resinous substances has been gaining attention. Bisphenol A (BPA), present in resinous materials, has shown the potential to reach significant concentrations in wastewater streams, the environment, the human body, and even urine (Reidelbach et al. 2021). Examples of these effects include the release of BPA during the removal of old restorations, the adjustment of new restorations, the process of manufacturing indirect restorations (through milling and grinding), and the production of resin‐based dental prostheses (Binner et al. 2022; Cokic et al. 2017; Mulligan et al. 2021; Polydorou et al. 2020; Talsness et al. 2009).

In addition to dental materials, DWW contains human secretions, including saliva, blood, and tissues. When using the dental suction system and spitting into the equipment called the “dental cuspidor bowl,” a complex network of water lines (plastic pipes) becomes contaminated with high densities of microorganisms, primarily gram‐negative environmental bacteria. This contamination has the potential to include pathogens (Kanamori et al. 2024). Ultimately, these microorganisms are discharged into the sewage, potentially causing imbalances in human and environmental ecosystems, highlighting DWW as remarkably biodiverse (Bristela et al. 2012; Rani et al. 2015). Furthermore, the interaction between heavy metals and microbes in dental sewage can increase selection pressure, potentially leading to the emergence of new antimicrobial determinants and antimicrobial resistance (Jiao et al. 2023).

In the quest to advance techniques for preventing toxic effluents, the filtration of amalgam waste is gaining visibility and can be employed in dental offices worldwide (Olivera et al. 2020). Furthermore, environmental regulators in the United States are consistently mandating the installation of amalgam separators that comply with ISO 11143 standards in dental clinics. This movement reflects efforts to reduce the impacts of effluents. However, when compared to particles from metal‐free materials, the filtration of DWW has shown efficacy, presenting a promising approach for the treatment of dental effluents (Polydorou et al. 2020).

Global efforts toward sustainable environmental practices underscore the urgent need for a comprehensive risk assessment regarding the waste generated from materials used daily in dental offices during procedures. Due to the scarcity of evidence in this field, we propose conducting a scoping review study. This type of study will allow us to comprehensively map and analyze the information already published in the literature on this topic, providing valuable insights for future research in the area. The primary objective of this study was to comprehensively map the available evidence in the literature on the chemical, physical, and microbiological risks associated with DWW, both for the environment and human health. Additionally, we aimed to describe potential strategies and treatment methods focusing on reducing risks related to the disposal of these DWWs.

## 2 Methods

This study is designed as a scoping review. The study protocol is available on the Open Science Framework platform via the link https://osf.io/dfknz/, with the identifier: DOI 10.17605/OSF.IO/DFKNZ. The final study report will follow the PRISMA‐ScR guidelines (Tricco et al. 2018).

### 2.1 Eligibility Criteria

All types of studies published in the form of scientific articles in journals addressing information about DWW, including its management, characterization, filtration, and associations with environmental and human health effects, were included in this analysis.

### 2.2 Consent

The consent of interest involves mapping the available evidence in the literature regarding the risks that DWW may pose to the environment and humans and cataloging possible strategies reported for reducing these risks, if reported.

### 2.3 Context

No restrictions were applied regarding the study's publication date. However, due to funding constraints, only studies published in the English language were included.

### 2.4 Types of Evidence Sources

It included articles of any study design, such as experimental, laboratory, observational studies, and reviews.

### 2.5 Strategies of Search

The search strategy was developed by researchers based on PubMed MeSH terms, entry terms, and free terms. The search was initially conducted on PubMed and later adapted to the Scopus and Web of Science databases (Frame 1). No time restrictions were applied to the searches. The last search was carried out on June 22, 2024.

**Table:** 

Source of information	Search conducted
Pubmed	**(((((((((((((((((“Waste Water”[Mesh]) OR (“Waste Disposal, Fluid”[Mesh])) OR (“Dental Waste”[Mesh])) OR (Dental unit water)) OR (Waste Water)) OR (Wastewater dental)) OR (Waste)) OR (Wastes)) OR (Waste Dental)) OR (Wastewater)) OR (Dental wastewater)) OR (Wastewater)) OR (Waste water)) OR (DWW)) OR (dental‐unit wastewater)) OR (Dental wastewater)) OR (waste streams)) AND (((((((((((((“Dentistry”[Mesh]) OR (“Dental Equipment”[Mesh])) OR (“Dental Offices”[Mesh])) OR (“Dental Clinics”[Mesh])) OR (dental practices)) OR (Dentistry)) OR (dental offices)) OR (Dental Office)) OR (Dental Practice)) OR (dental chair)) OR (dental Chairside)) OR (dental clinic)) OR (Dental clinics))**
Scopus	(Utilized filter: article title, abstract, and keywords) **‘** **Dental unit water’ OR ‘Waste Water OR ‘Wastewater dental’ OR ‘Waste’ OR ‘Wastes' OR ‘Waste Dental’ OR ‘Wastewater’ OR ‘Dental wastewater’ OR ‘Wastewater’ OR ‘Waste water’ OR ‘DWW’ OR ‘dental‐unit wastewater’ OR ‘Dental wastewater’ OR ‘waste streams' AND ‘Dentistry’ OR ‘Dental Equipment’ OR ‘Dental Offices' OR ‘Dental Clinics' OR ‘dental practices' OR ‘Dentistry’ OR ‘dental offices' OR ‘Dental Office’ OR ‘Dental Practice’ OR ‘dental chair’ OR ‘dental Chairside’ OR ‘dental clinic’ OR ‘Dental clinics’**
Web of Science	**(TS =** **(“Waste Water”[Mesh]) OR (“Waste Disposal, Fluid”[Mesh])) OR (“Dental Waste”[Mesh])) OR (Dental unit water)) OR (Waste Water)) OR (Wastewater dental)) OR (Waste)) OR (Wastes)) OR (Waste Dental)) OR (Wastewater)) OR (Dental wastewater)) OR (Wastewater)) OR (Waste water)) OR (DWW)) OR (dental‐unit wastewater)) OR (Dental wastewater)) OR (waste streams)) AND (((((((((((((“Dentistry”[Mesh]) OR (“Dental Equipment”[Mesh])) OR (“Dental Offices”[Mesh])) OR (“Dental Clinics”[Mesh])) OR (dental practices)) OR (Dentistry)) OR (dental offices)) OR (Dental Office)) OR (Dental Practice)) OR (dental chair)) OR (dental Chairside)) OR (dental clinic)) OR (Dental clinics))**

**Frame 1:** Search Strategy.

### 2.6 Study Selection

The study selection was carried out using the Rayyan QCRI program (https://rayyan.qcri.org), where duplicate removal was performed. Initially, a pilot test was conducted to assess agreement in the selection of studies between the two reviewers involved in this phase. Two researchers, independently, identified articles by analyzing titles and abstracts for relevance and the presence of eligibility criteria. These articles were classified as “included,” “excluded,” or “maybe.” Subsequently, articles classified as included and may have were chosen for full‐text reading and additional eligibility screening by the same two reviewers, also independently. Discrepancies in the selection of titles/abstracts and full‐text articles were resolved through discussion. In case of disagreement, the opinion of a third reviewer was sought.

### 2.7 Data Collection

A standardized data extraction form was created using the Excel program (Microsoft Excel for Mac, Microsoft—Tables 1, 2, and 3). Initially, 10 included studies were selected to test data extraction to ensure consistency in the interpretation of items. A pilot test was conducted through discussions among the three reviewers involved to review and discuss the data to be extracted. Subsequently, two reviewers independently extracted data from half of the included studies (G.P.F., R.M.B.), and a third reviewer checked the consistency of the data (L.R.).

**TABLE 1:** Characteristics of the studies included in the study (*n* = 29).

Characteristics	*n* (%)
Study design	
Experimental	16 (55.1%)
Laboratory	9 (31.3%)
Clinical and experimental	1 (3.4%)
Prospective longitudinal (observational design)	1 (3.4%)
Systematic review	1 (3.4%)
Literature review	1 (3.4%)
Sample component:	
DWW from the dental chair units after dental procedure	18 (62.1%)
Simulated dental wastewater using metal‐free materials	3 (10.3%)
Simulated dental wastewater with materials based on dental amalgam	5 (17.2%)
Simulated dental wastewater with microorganisms	1 (3.4%)
Studies	2 (6.9%)
Key parameters analyzed within the studies:	
Analysis of metals (mainly mercury)	18 (62.0%)
Microbiological analysis	6 (20.6%)
BPA analysis	2 (6.9%)
Other parameters: physicochemical (pH, specific conductivity, total suspended solids [TSS], and total dissolved solids [TDS])	7 (24.1%)
Cytotoxicity	1 (3.4%)
Ecotoxicity	1 (3.4%)
Estrogenicity	1 (3.4%)
Not evaluated (reviews)	2 (6.9%)
Reports environmental risks of dental wastewater?	
Yes	22 (75.8%)
Not reported	7 (24.1%)
Reports risks of dental wastewater to humans?	
Yes	5 (17.2%)
Not reported	24 (82.7%)
Reports strategies to reduce environmental damage from dental wastewater?	
Yes	22 (75.8%)
Not reported	7 (24.1%)

**TABLE 2:** Outcomes and results of included studies.

		Study design	Sample	Objective	Methods	Results
1	Arenholt‐Bindslev and Larsen 1996	Experimental	DWW	Obtain data on the amount of mercury (Hg) improperly disposed of in the wastewater from dental clinics.	Hg content in the samples was determined by cold vapor atomic absorption spectrophotometric analyses (CV‐AAS) according to SM 303F	Hg is released with wastewater from dental clinics. The average discarded Hg value in 10 clinics without an amalgam separator was 250 mg per dentist (range from 65 to 842). In clinics equipped with an amalgam separator, the average value was 35 mg Hg per dentist (range from 12 to 99).
2	Pederson et al. 1999	Experimental	DWW	Testing the ability of two such polymers, individually and in combination, to remove Hg from dental operatory wastewater	The method used to assess Hg levels was CV‐AAS.	Total Hg from the five continuously mixed samples ranged from 5.3 to 12.0 mg/L (mean = 7.3 mg/L, standard deviation [SD] = 2.74). A combination of the two polymers can sufficiently reduce Hg levels to allow discharge of DWW into public sewer systems. Hg from dental surgeries in wastewater has the potential for environmental risk.
3	Chin et al. 2000	Literature review	Studies reporting the environmental impact of dental amalgam, with reference to the effects attributed to its Hg content.	Describe impact of dental amalgam will be discussed, with reference to the effects attributed to its Hg content.	Not reported	Data from the studies reviewed show that dental clinics equipped with amalgam separators in the dental chair demonstrated a lower average Hg level (10%). The environmental impact of dental Hg is due to poor management of amalgam waste, not just what is released through sewage. The use of amalgam separation devices can reduce the amount of amalgam‐contaminated water released into dental clinics.
4	Adegbembo et al. 2002	Laboratory	Replicas of artificial teeth and natural teeth containing amalgam restorations (simulated DWW).	To determine the amount of amalgam entering the waste stream during removal of dental amalgam restorations	Hg concentration was measured with a VGA 77 cold vapor system and a Spectra 880 atomic absorption spectrophotometer	About 60% of the amalgam was found in DWW. About a third was retained in the primary solids separator and less than 10% was retained in the secondary solid's separator. A high concentration of Hg (average 31.2973 mg/L) was found in the wastewater samples when the ISO‐certified separator was not connected to the dental chair.
5	Fan et al. 2002	Experimental	Slurry composed of the amalgam particles and 1 L of filtered (1‐μm nominal pore size) water containing 1 g of sodium pyrophosphate.	To evaluate the amalgam removal efficiency by commercially available amalgam separators and the total Hg concentration in the simulated DWW.	For the determination of total Hg, EPA Method 245.1 was used through CV‐AAS	12 available amalgam separators had amalgam removal efficiencies of 96% or greater.
6	Kennedy 2003	Experimental	Dechlorinated water and dental amalgam (simulated DWW)	To determine if Hg was biologically available for uptake and accumulation in the common goldfish, *C. auratus* during an environmental exposure to dental amalgam.	Sampled fish were randomly coded, and then dissected and digested according to standard procedures (EPA, 1991). The liver, brain, and a subsample of muscle from the right midline of the fish (skin removed). The final solutions were analyzed for total Hg by cold vapor atomic fluorescence spectroscopy (particle size analysis [PSA] Millenium Merlin System 10.025, PS Analytical, UK)	Representative samples of dental amalgam found in the dental wastewater discharge stream are bioavailable to fish and can accumulate in the internal tissues of the fish *C. auratus* .
7	Reed et al. 2002	Experimental	DWW	To determine if placement of a membrane separation system at the WVU Dental Clinic is warranted. Testing two membranes, Hollow fiber (HF) or tubular.	Hg concentrations were determined using the USEPA Method 245.1, based on CV‐AAS, with a Perkin Elmer FIAS 100/3100 system. Solid‐phase samples were digested according to USEPA Method 7470A prior to Hg analysis. Additional physicochemical analyses were performed, including pH (Orion 520A meter), conductivity (Accuret 30), and turbidity (HF Scientific 2000).	Suspended solids concentration was reduced by 25%, total Hg concentration decreased by about 50%. The membranes had similar Hg rejections (tubular:97%, HF:98%).
8	Stone et al. 2003	Experimental	DWW	To establish whether monomethyl Hg (MeHg) is present in wastewater from dental units and, if present, determine the concentration relative to total Hg.	Total Hg was measured using EPA Method 1631 (BrCl oxidation, reduction, gold trap, CVAF detection). MeHg was analyzed via modified EPA Method 1630 (solvent extraction, ethylation, Tenax trap, GC‐CVAFS). A combined method for MeHg, ionic Hg, and total Hg in biological samples has also been described.	Environmentally important levels of MeHg are present in dental wastewater at concentrations orders of magnitude higher than those observed in natural environments such as oceans, lakes, and rain.
9	Reed et al. 2004	Experimental	DWW	Not specified in the article. Authors understand that it is necessary to evaluate which behaved in fashion filtering membrane is more effective, HF or tubular.	Hg levels were determined using EPA Method 245.1, with cold vapor atomic absorption (Perkin Elmer FIAS 100/3100). Solid‐phase samples were digested per EPA Method 7470A. pH (Orion 520A), conductivity (Accuret 30), and turbidity (HF Scientific 2000) were measured in permeate and distilled water samples.	Both membranes rejected the vast majority of Hg (99%). The system can be operated indefinitely, but biological growth has blocked the lumen openings of a type of HF membrane, requiring frequent maintenance.
10	Batchu et al. 2006	Experimental	Cylindrical amalgam specimens (simulated DWW)	Evaluation of 47 disinfectants or line cleaners for their potential to release Hg from amalgam waste	The Hg content was determined using a modified EPA Method 245.1, which involves CV‐AAS. The pH of each solution was measured using an Accumet pH meter.	Six disinfectants released significantly more Hg from the amalgam than the control, which was deionized water. Preparations containing chlorine release more Hg from amalgam than some other products and the deionized water control.
11	Stone et al. 2008	Experimental	DWW	Evaluated the ability of a chairside low‐cost filtration system to remove particulate‐based Hg from dental‐unit wastewater.	Hg concentrations were determined with CV‐AAS (USEPA method 7470A).	Demonstrated that low‐cost filtration systems can function as effective amalgam separators, removing substantial amounts of Hg‐containing amalgam waste without degrading the chairside vacuum levels. System demonstrated 99.6% removal efficiency in a clinical setting, and 96.8% efficiency when evaluated in the laboratory.
12	Zhao et al. 2008	Experimental	DWW	The DWW was characterized to determine whether methyl Hg generation occurred and to identify conditions affecting the observed results.	The characterization of dental wastewater (DWW) included measuring pH with a probe after sample homogenization. Total suspended solids and volatile suspended solids were determined following Standard Methods. Dissolved organic carbon and total organic carbon were analyzed in both filtered and unfiltered samples using elemental analysis. Anions were quantified by ion chromatography. Sulfide concentrations in filtered and unfiltered samples were preserved and measured using a modified iodometric titration. Microbial DNA was extracted using a commercial protocol optimized for the complex DWW matrix, and quantitative PCR (qPCR) was performed to quantify total eubacteria and sulfate‐reducing bacteria (SRB). Total Hg and MeHg concentrations were determined after oxidative digestion using cold vapor atomic fluorescence spectroscopy	Methyl Hg is present at high levels in DWW. Highly significant correlations were found between methyl Hg and both amplified Desulfobacteraceae and Desulfovibrionacaea DNA, both of which are known Hg methylaters.
13	Hamza et al. 2010	Experimental	DWW	Synthesize and characterize a novel reagent 6‐hydroxy‐3‐(2‐oxoindolin‐3‐ylideneamino)‐2‐ thioxo‐2H‐1,3‐thiazin‐4(3H)‐one. Develop an accurate method for the analysis of Hg in different wastewater samples.	A spectrophotometric method was developed for the sensitive determination of traces of Hg (II) ions in samples. The method is based on the formation of a stable complex between Hg (II) ions and the chromogenic reagent 6‐hydroxy‐3‐(2‐oxoindolin‐3‐ylideneamino)‐2‐thioxo‐2H‐1,3‐thiazin‐4(3H)‐one (HOTT), with maximum absorption at 505 nm in Britton‐Robinson buffer (pH 4–6). The corrected absorbance of the complex was obtained by means of the spectrophotometric correction method, obeying the Beer–Lambert Law in the concentration range of 0.2–2.0 μg mL^−1^ and Ringbom graphs between 0.32 and 0.96 μg mL^−1^.	Traces of Hg were found in the wastewater. The proposed method for analyzing Hg in wastewater was accurate (95%) and validated. This method is a simple, reliable and low‐cost means and can be used for the rapid and accurate determination of trace amounts of Hg in DWW samples.
14	Shraim et al. 2011	Experimental	DWW	Quantify the amount of soluble Hg in the wastewater of some dental clinics in Al‐ Madinah Al‐Munawarah (KSA) and to examine if any of the other dental amalgam constituents is found in the clinics' wastewater	As amostras foram analisadas em duplicata, uma para determinação de mercúrio (Hg) e outra para análise de outros metais, utilizando diferentes parâmetros experimentais. A determinação de íons mercúrio (II) foi realizada por espectrofotometria UV–Vis, baseada na formação de um complexo colorido com o reagente cromogênico 6‐hidroxi‐3‐(2‐oxoindolin‐3‐ilidenoamino)‐2‐tioxo‐2H‐1,3‐tiazin‐4(3H)‐ona (HOTT), com leitura da absorbância máxima a 505 nm em tampão Britton‐Robinson (pH 4–6). O reagente HOTT foi sintetizado via condensação direta da isatina com hidrazida de ácido fórmico ditióico, seguida de refluxo e purificação para garantir sua pureza e eficiência na reação. Para análise quantitativa precisa, tanto do mercúrio quanto de outros metais, utilizou‐se a espectrometria de massa com plasma indutivamente acoplado (ICP‐MS)	It is evident that most samples contain Hg concentrations much higher than the permissible value for the source water discharged into public sewer. Most of the samples were found to contain hazardous levels of metals, especially Mg, Mn, Cu, Zn, Sn, Ba, and Hg, many of which are amalgam constituents.
15	Bristela et al. 2012	Experimental	DWW	Evaluate whether the counts of aerobic heterotrophic bacteria correlate with the presence of potentially pathogenic bacteria such as *Legionella* spp. or *Pseudomonas aeruginosa*	The water samples were tested for aerobic heterotrophic colony‐forming units using the pour plate method with amounts of 1 mL and 1 mL of samples at 1:100 dilution. For the detection of *P. aeruginosa* and *Legionella* spp. the membrane filter method was used.	For DWW waters, *Legionella* spp. could be found in 39 samples. *P. aeruginosa* was detected in one sample.
16	Zhao et al. 2012	Experimental	DWW	Determine whether Hg methylation specifically from DWW (not domestic activated sludge) can occur in the aeration	pH was measured using a pH probe (Fisher Scientific, Fairlawn, NJ) calibrated with three standard solutions with pH values of 4, 7, and 10. Temperature and dissolved oxygen (DO) were measured using a combined DO probe with temperature sensor (BLD Science, Raleigh, North Carolina). Total Hg and MeHg concentrations were determined after oxidative digestion using cold vapor atomic fluorescence spectroscopy	Although some Hg methylation may occur in the sewer collection system, additional methylation is unlikely to occur in the aeration basin in activated sludge wastewater treatment plants.
17	Oliveira et al. 2014	Clinical and experimental	10 patients and DWW sample	Discover the potential for Hg contamination of amalgam in patients and dental wastewater.	To assess patients' Hg levels, urine was collected immediately before the restorative procedure and 48 h after. CV‐AAS was used as the urine testing method.	Increased levels of Hg contamination were found in patients. The potential for contamination of Hg‐containing wastewater was significant.
18	Rani et al. 2015	Experimental	DWW	Detailed assessment of the DWW microbiome impacted with high levels of tHg, MeHg, and potentially antibacterial heavy metals like Ag, Zn, and Cu. A central aim is also to identify the role microorganisms play in DWW samples with and without high levels of MeHg, in particular focusing on the role known Hg methylators like SRB may play in Hg methylation.	Samples for metal analysis were prepared according to USEPA method SW 3050B and all metals except Hg were analyzed by inductively coupled plasma‐mass spectrometry using USEPA method SW 6020A. Total Hg was quantified by USEPA standard method 1631 and 163,060 using a Brooks–Rand Cold Vapor Atomic Fluorescence Spectrometry (CVAFS) System (model III, Brooks–Rand, Seattle, WA). DNA extraction and automated ribosomal intergenic spacer analysis (ARISA) of DWW microbiome.	High concentrations of Hg, MeHg, and heavy metals were detected in DWW. The microbiome was dominated by Proteobacteria, Actinobacteria, Bacteroidetes, Chloroflexi, and many unclassified bacteria. Significant correlations were found between the bacterial community, Hg levels, and geochemical factors. Microbial communities are complex and highly dynamic, strongly correlate with MeHg levels, and geochemical data are consistent with SRB‐mediated methylation in DWW.
19	Cataldi et al. 2017	Systematic review	Eight studies	To systematically study the incoming dental unit water and the waste one, focusing the attention on the problem of the wastewater contamination and its regulations.	Systematic review of the literature of the last 17 years on the topic of effluents from dental units. Italian and English were the languages chosen for the research of the articles.	No research was found on the composition of effluents from dental units, with the exception of dental amalgam. There is a lack of scientific evidence demonstrating that effluents from dental units are dangerous
20	Ghasemi and Masoudirad 2020	Experimental	DWW	To evaluate heavy metal concentrations in wastewater of the Faculty of Dentistry, Shahid Beheshti University of Medical Sciences, Tehran, Iran.	Concentrations of Hg, lead, cadmium, nickel, and copper were measured using direct air‐acetylene flame atomic absorption spectrometry by a Hach	Although the practice of dentistry at this school adds some heavy metals to the environment, the amount is still below permitted levels for lead, copper, cadmium, nickel; however, we need to have more control over the safe disposal of Hg.
21	Olivera et al. 2020	Experimental	DWW	To evaluate the performance of a commercially available amalgam separator in a clinical setting in which a relatively high number of amalgam restorations are performed.	Total Hg concentration by inductively coupled plasma mass spectrometry (ICP‐MS)	Hg concentrations in the effluent ranged from 0.05 to 11.93 mg/L. The total solids accumulated at the end of the separator's useful life was 195.4663.4 g. Separator performance varies depending on a variety of clinical factors in addition to the amount of amalgam used in practice.
22	Polydorou et al. 2020	Experimental	Three resin composites (Ceram X, Filtek Supreme XTE, and Core‐X flow‐simulated DWW)	To evaluate the release of bisphenol A (BPA) in wastewater after grinding of resin composites and tested three filtration materials.	For reliable qualitative and quantitative determination of BPA, high‐performance liquid chromatography–fluorescence detector analysis was performed.	The detected amount of BPA in the Ceram X and Filtek Supreme XTE resin group was significantly higher than that in the (Core‐Xflai) group. BPA represents an important hazardous substance in composite resin eluates due to its estrogenic effects even in small doses. BPA is released into wastewater through the wear of dental composites.
23	Scarano et al. 2020	Experimental	Sterile distilled water and microbiological contamination (simulated DWW)	To investigate the capacity of a medical device of ultrafiltration for dental units to filtrate coxsackieviruses.	The determination of the microbial load at 37°C on plate count agar was performed: the contaminated physiological sample was inoculated in cell cultures to verify initial viral titer. The filtered physiological sample was inoculated in cell cultures to determine residual viral load. Viral titer was established according to the Reed–Muench method, and was expressed in TCID50/25 μL.	The filters showed an effective retention capacity of the viral loads under examination, always recording a residual zero load, even in the presence of high initial contaminant loads with coxsackievirus B5 used and in every case, demonstrating a reduction of 99.9999% in the volume analyzed.
24	Mulligan et al. 2021	Experimental	Standardized discs particulate resin‐based composite (RBC) waste	Characterize various examples of dental RBC microparticulate waste following release into the environment by means of PSA. To assess the potential reactivity of the RBC microparticles in the environment and their potential to become toxic polluters and/or vectors for pollution.	The techniques utilized for the characterization of RBC microparticles in wastewater samples were scanning electron microscopy (SEM), laser diffraction PSA, micro‐Fourier transform infrared (FTIR) spectroscopy and potentiometric titration.	RBC microparticle waste is a small contribution to environmental pollution when compared to plastic microparticle waste from other industries.
25	Reidelbach et al. 2021	Experimental	Four composite resins (simulated DWW).	Evaluated the cytotoxic and estrogenic effects of dust and eluates released into simulated wastewater after grinding of dental resin‐based materials	The study used three main methods to evaluate the toxicity of substances related to dental materials. First, the test with the luminescent bacterium *Vibrio fischeri* was applied, according to the ISO 11348‐3:2009 standard, in which the inhibition of light emission by the bacteria indicates the presence of toxic compounds in the eluates of materials, dental monomers, and BPA dissolved in water. Next, the WST‐1 assay was used with A549 human lung epithelial cells, which measures cell viability through mitochondrial activity, evaluating the conversion of the WST‐1 reagent into colored formazan, which allows inferring the cytotoxic potential of the samples. Finally, the lacZ assay was used to analyze the activity of the enzyme β‐galactosidase as an additional functional marker, with readings made by spectrophotometry after standardized incubation.	Toxic effects on bacteria and human cells (bactericidal and cytotoxic effects in vitro) were verified. BPA induced an estrogenic effect.
26	Binner et al. 2022	Experimental	DWW	Measuring particle load and toxicity of amalgam‐free dental materials in DWW	The ecotoxicity test was performed by immobilizing *Daphnia magna* . To evaluate the particle size, SEM (scanning electron microscopy) analysis was performed.	High Ph and conductivity were detected in DWW. Particles of the material are detected in wastewater streams. The toxicity of DWW was confirmed. Changed parameters have the potential to cause environmental impact.
27	Jiao et al. 2023	Experimental	DWW	To investigate the abundance and components of bacterial communities, antibiotic resistance genes (ARGs), and mobile genetic elements (MGEs) in treated and untreated dental wastewater using advanced metagenomic methods. The study aimed to better understand the evolution of antimicrobial resistance in a heavy metal environment	The study employed metagenomic DNA extraction from sewage sediments, followed by quantification and library construction for sequencing on the Illumina platform. Sequences were pre‐processed for quality control and contig assembly, and ARGs and MGEs were identified using the CARD, ResFinder, and a specific MGE database. Statistical analyses included alpha and beta diversity (using Shannon and Simpson indices and PCoA), as well as co‐occurrence correlations between ARGs, MGEs, and microorganisms through network analysis using Spearman's correlation coefficient (*ρ* ≥ 0.7; *p* < 0.001).	The main results indicated that dental wastewater contained a significant abundance of ARGs and MGEs, contributing to the occurrence and spread of antimicrobial‐resistant bacteria (ARB). A total of 1208 types of ARGs and 93 MGE subtypes were identified. Treatment of wastewater with membranes and ozone was only partially effective in removing some species of bacteria and types of ARGs and MGEs.
28	Albishri and Yakout 2023	Experimental	DWW (Hg íons simulate)	To develop a low‐cost thio‐functionalized biochar from cape gooseberry leaves for the efficient removal of Hg ions from dental effluents, aiming to reduce environmental Hg contamination.	Hg concentrations were determined with CV‐AAS (USEPA method 7470A)	The main results indicated that thio‐functionalization significantly improved the biochar's affinity for Hg uptake by 54%. The Hg removal efficiency was 96.4% at pH 5.0 with 25 min of contact time. The modified biochar reduced Hg concentration in dental effluents to below 10 parts per billion.
29	Kanamori et al. 2024	Prospective longitudinal	DWW	To monitor trends of asymptomatic SARS‐CoV‐2 infection in children by analyzing wastewater from a pediatric dental clinic	Viral RNA concentration, extraction, and purification Viral RNA was concentrated using the polyethylene glycol precipitation method. Real‐time reverse transcription PCR (RT‐PCR) SARS‐CoV‐2 RNA was detected using the SARS‐CoV‐2 Detection RT‐qPCR Kit for Wastewater (Takara Bio, Shiga, Japan) and QuantStudio 5 Real‐Time PCR System (Thermo Fisher Scientific, Waltham, MA, USA)	The main results indicated that the presence of SARS‐CoV‐2 RNA in the clinic's wastewater was significantly associated with the number of weekly COVID‐19 cases in the local pediatric population. Viral RNA detection coincided with the COVID‐19 waves associated with the Delta and Omicron variants but not with the Alpha variant.

**TABLE 3:** Characteristics of studies and strategies to mitigate environmental impacts.

Study	Country	Strategy for reducing environmental risks
Arenholt‐Bindslev and Larsen 1996	Denmark	Recommends the installation of efficient amalgam separators to significantly reduce mercury (Hg) intake.
Pederson et al. 1999	EUA	Suggests that with the current information, more suitable automated treatment methods can be developed for use in smaller dental offices.
Chin et al. 2000	Literature Review	Suggests the use of amalgam separation devices on dental chairs to reduce the amount of water contaminated with released amalgam in dental clinics.
Adegbembo et al. 2002	Canadá	Suggests, as the most logical standard for all of Canada, the voluntary installation of amalgam particle separators in dental offices.
Fan et al. 2002	EUA	Recommends the use of separators on dental chairs to reduce the total Hg concentration in the wastewater.
Reed et al. 2002	EUA	Recommends the use of a membrane filtering system on the dental chair that is operationally simple to increase the likelihood of acceptance by small dental offices.
Kennedy 2003	Canada	Not reported.
Stone et al. 2003	EUA	Suggests the installation of systems to remove Hg from dental unit wastewater.
Reed et al. 2004	EUA	Suggests that the use of tubular membranes would be effective in dental chairs.
Batchu et al. 2006	EUA	Recommends that dental professionals avoid using disinfectants for treating dental unit water lines.
Stone et al. 2008	EUA	Recommends the use of low‐cost and effective filtration systems on dental chairs for the removal of substantial amounts of Hg.
Zhao et al. 2008	EUA	Not reported.
Hamza et al. 2010	Saudi Arabia	Suggests the use of more affordable methods for Hg determination so that dental clinics can adhere and have better access.
Shraim et al. 2011	Saudi Arabia	Not reported.
Bristela et al. 2012	Austria	It is strongly recommended that distribution piping and dental wastewater be tested for *Legionella* spp. and *Pseudomonas aeruginosa* to reduce infections by clinically relevant pathogens.
Zhao et al. 2012	EUA	Recommends better dental clinic management practices to limit Hg at the source, rather than controlling it after it is diluted by the much larger quantities of domestic and commercial/industrial wastewater. Therefore, the control and/or elimination of Hg from DWW can be better achieved through control at the source and not at the WWTP stage.
Oliveira et al. 2014	Brazil	Not reported.
Rani et al. 2015	EUA	Suggests the need to broaden our understanding of Hg methylation and bacterial species' resistance to the metal.
Cataldi et al. 2017	Italy	Suggests improving national laws.
Olivera et al. 2020	EUA	It suggests the use of amalgam separators in the dental chair that are considered competitive products with low cost and installation versatility.
Polydorou et al. 2020	Germany	Use an DWW filtration method to remove BPA. Mainly the filtration method composed of catalytic carbon (activated carbon) appears to be an effective method.
Scarano et al. 2020	Italy	It suggests the use of filters in the dental chair that are effective in retaining viral loads.
Ghasemi and Masoudirad 2020	Iran	Not reported.
Reidelbach et al. 2021	Germany	Not reported.
Mulligan et al. 2021	England	Not reported.
Binner et al. 2022	Ireland	Suggests the use of filters in the dental unit chair to filter new materials, not just amalgam.
Jiao et al. 2023	China	Not reported.
Albishri and Yakout 2023	China	The use of thiol‐functionalized biochar from cape gooseberry leaves as an innovative and potentially low‐cost strategy for removing Hg ions (Hg (II)) from dental wastewater.
Kanamori et al. 2024	Japan	Not reported.

The following data were extracted: study and year, study design (experimental studies, laboratory studies, reviews, randomized clinical trials, observational studies, or others), country where the study was conducted, sample extraction site (office, clinics with multiple chairs, universities, or other), sample components (dental office wastewater, laboratory‐simulated dental office wastewater, or other), study objective, parameters analyzed for characterization (reported physicochemical parameters), and main results. For the outcome, it was noted whether the article mentioned environmental risks, posed risks to humans, and whether strategies to minimize environmental damage were described.

### 2.8 Data Analysis

A descriptive analysis of the data was conducted, considering the following aspects: study design, components of each sample, parameters analyzed for characterization, as well as environmental risks (categorized as “yes,” “no,” or “not reported”) and risks to humans (categorized as “yes,” “no,” or “not reported”). The strategies adopted to reduce environmental damage were also examined and classified as yes or no. The VOSviewer tool was used to visualize and analyze the data. This software specializes in bibliometrics, allowing for the mapping and interpretation of scientific collaboration networks. This tool was used to analyze and visualize connections between authors, as well as the co‐occurrence of keywords.

## 3 Results

**FIGURE 1:**
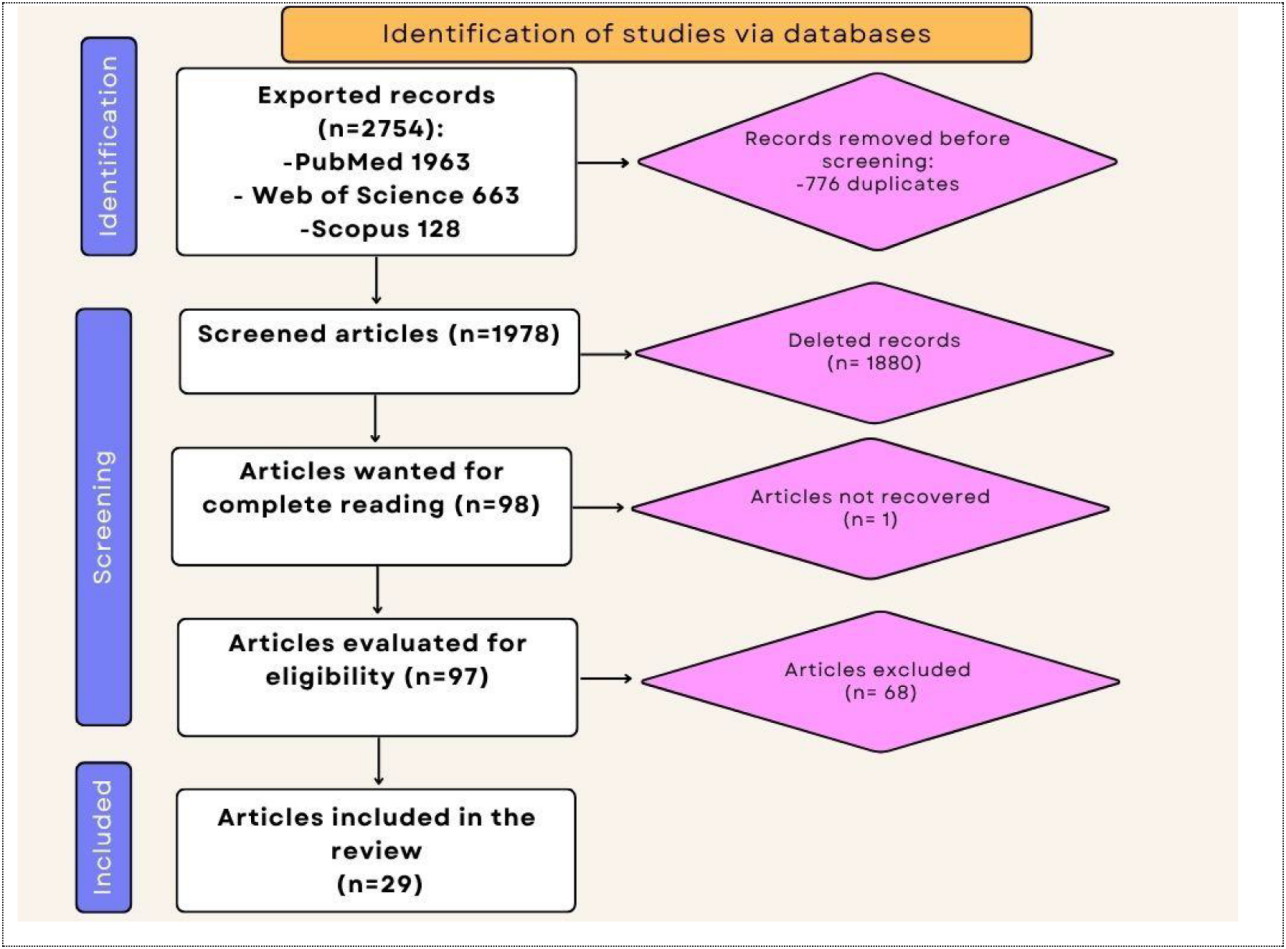
Study selection flowchart.

The search in the selected databases resulted in 1963 records exported from PubMed, 663 from Web of Science, and 128 from Scopus. After identifying 776 duplicates, these were excluded, leaving 1847 articles. After analyzing titles and abstracts, 1978 articles were discarded, resulting in 98 articles eligible for full evaluation. Of the 98 reports assessed, one article could not be accessed in full, despite attempts to obtain it through emails to the authors. Thus, 97 articles had their full texts analyzed. After a detailed review, 68 of these were excluded, resulting in the inclusion of 29 studies in this scoping review. Figure 1 presents the flowchart of the study selection process. A complete list of excluded studies, along with justifications, is available at the link https://osf.io/dfknz/.

Table 1 summarizes the main characteristics of the 29 studies included in this scoping review. Most of the studies (55.1%) adopted an experimental design, and 62.1% analyzed DWW collected directly from dental chair units after clinical procedures. Heavy metals, especially Hg, were the most frequently analyzed contaminants (62.0%). Additionally, 75.8% of the studies reported the polluting potential of DWW for the environment, and the same proportion discussed strategies to mitigate its environmental impact. However, only 17.2% of the studies addressed potential risks to human health.

Table 2 presents data from the analyzed samples, the central objective of the study, and the main results. A significant part of the studies (*n* = 18, 62.1%) have a central focus on the characterization of Hg or multimercury (MeHg) in the DWW (Adegbembo et al. 2002; Albishri and Yakout 2023; Arenholt‐Bindslev and Larsen 1996; Chin et al. 2000; Fan et al. 2002; Ghasemi and Masoudirad 2020; Hamza et al. 2010; Kennedy 2003; Oliveira et al. 2014; Olivera et al. 2020; Pederson et al. 1999; Rani et al. 2015; Reed et al. 2002, 2004; Shraim et al. 2011; Stone et al. 2003, 2008; Zhao et al. 2008, 2012). Of the 18 studies included, different methodologies were used. A study evaluated the amount of Hg present in fish tissues, finding significant levels (Kennedy 2003). Another study investigated the potential of disinfectants to increase Hg release rates, noting that six disinfectants resulted in significant additional Hg release (Batchu et al. 2006). A study evaluated the ecotoxicity of the effluent, confirming its environmental toxic potential (Binner et al. 2022). One of the articles reports the problems with national regulations in their countries (Cataldi et al. 2017) indicating that there is a lack of specific regulations and scientific evidence to make DWW dangerous. Six studies evaluated the microbiological risks of DW (Bristela et al. 2012; Jiao et al. 2023; Kanamori et al. 2024; Rani et al. 2015; Scarano et al. 2020; Zhao et al. 2008) Of these, one study investigated the composition of the microbiome present in DWW (Rani et al. 2015), while another examined the count of aerobic heterotrophic bacteria, in addition to clinically relevant pathogens, such as *Legionella* spp. and *Pseudomonas aeruginosa* (Bristela et al. 2012), and the most recent of these monitored the trends of asymptomatic SARS‐CoV‐2 infection in children by analyzing wastewater from a pediatric dental clinic (Kanamori et al. 2024). Eleven articles evaluated the use of filtration methods or separators for dental chairs (Albishri and Yakout 2023; Binner et al. 2022; Chin et al. 2000; Fan et al. 2002; Jiao et al. 2023; Olivera et al. 2020; Pederson et al. 1999; Polydorou et al. 2020; Reed et al. 2002, 2004; Scarano et al. 2020; Stone et al. 2008); highlighted among them, one of them evaluates the use of filters for resin‐based materials that are components of DWW (Polydorou et al. 2020). One of them evaluates the filtration of the coxsackievirus B5 virus, which may be present in the DWW and in the pipes of the dental chair, causing cross‐infection (Scarano et al. 2020). Another filtration method used in one of the studies was the treatment of DWW samples using a filter membrane and ozone (Jiao et al. 2023), while the others focus on the filtration of dental amalgam.

Table 3 presents the countries where the studies were carried out, as well as the articles that discussed strategies to mitigate environmental impacts, in addition to the main related strategies. Of the detailed studies, 11 articles (42.3%) were carried out in the United States, while the rest were carried out in different regions of the world. In South America, specifically, only one study (4%) was conducted by researchers in Brazil. Among the most frequently suggested strategies to reduce damage to the environment, the use of separators, filters, or filtration methods stands out, as indicated in 11 studies (37.9%).

**FIGURE 2:**
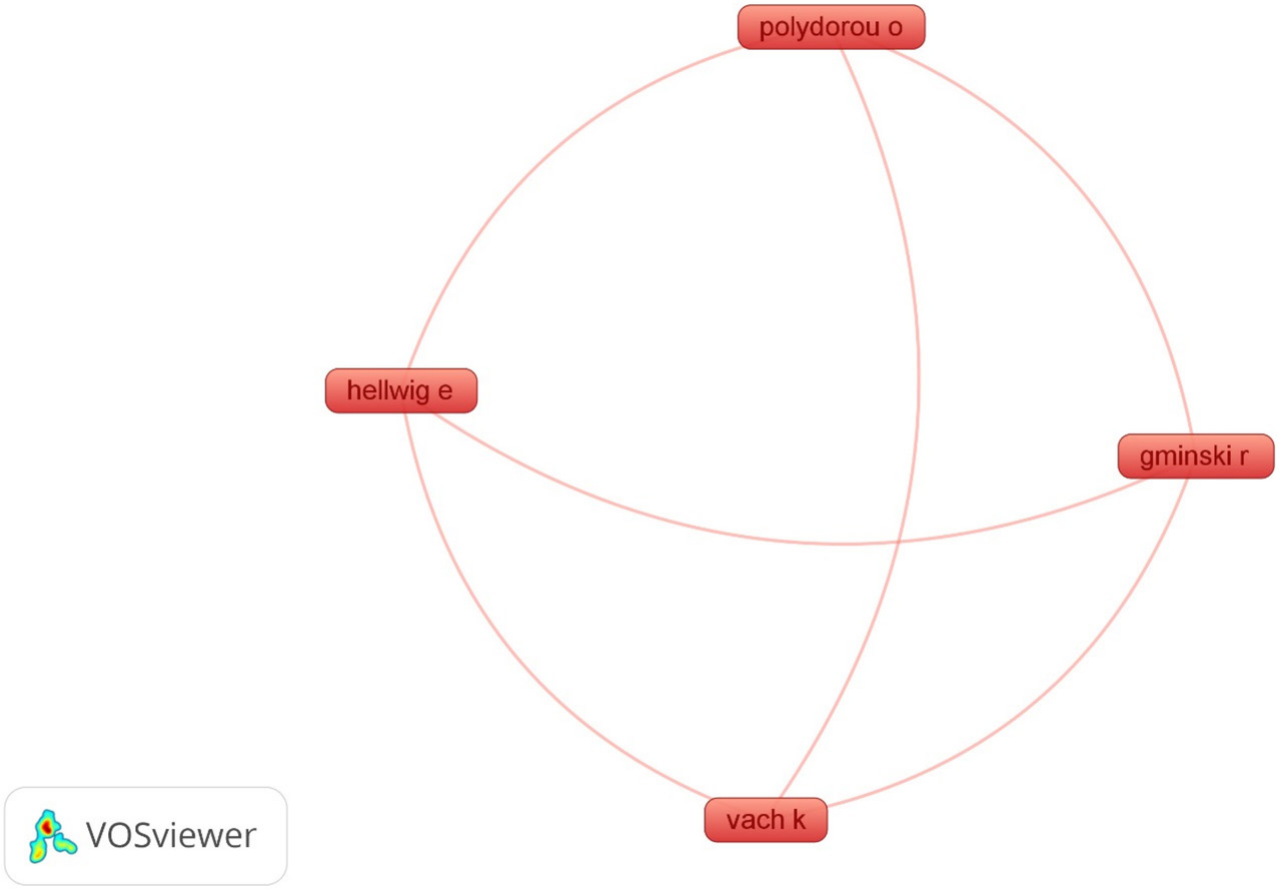
Cooperative relationships between authors in this field. The size of each node corresponds to the number of articles published by that author. The lines connecting the nodes represent the cooperative relationship between authors.

A total of 119 authors contributed to research in this area, with nine authors publishing two or more related studies. The authors with the highest number of publications are shown in Figure 2. Notably, the collaboration network among researchers exhibits significant distance, indicating low interconnectivity among the primary authors in the field. In Figure 2, the nodes represent the authors, and their size corresponds to the number of publications. The lines connecting the nodes indicate cooperative relationships between researchers. It is evident that, despite some specific collaborations, there is no highly connected core, suggesting that scientific production in this area may be fragmented into various research groups with minimal interaction between them.

**FIGURE 3:**
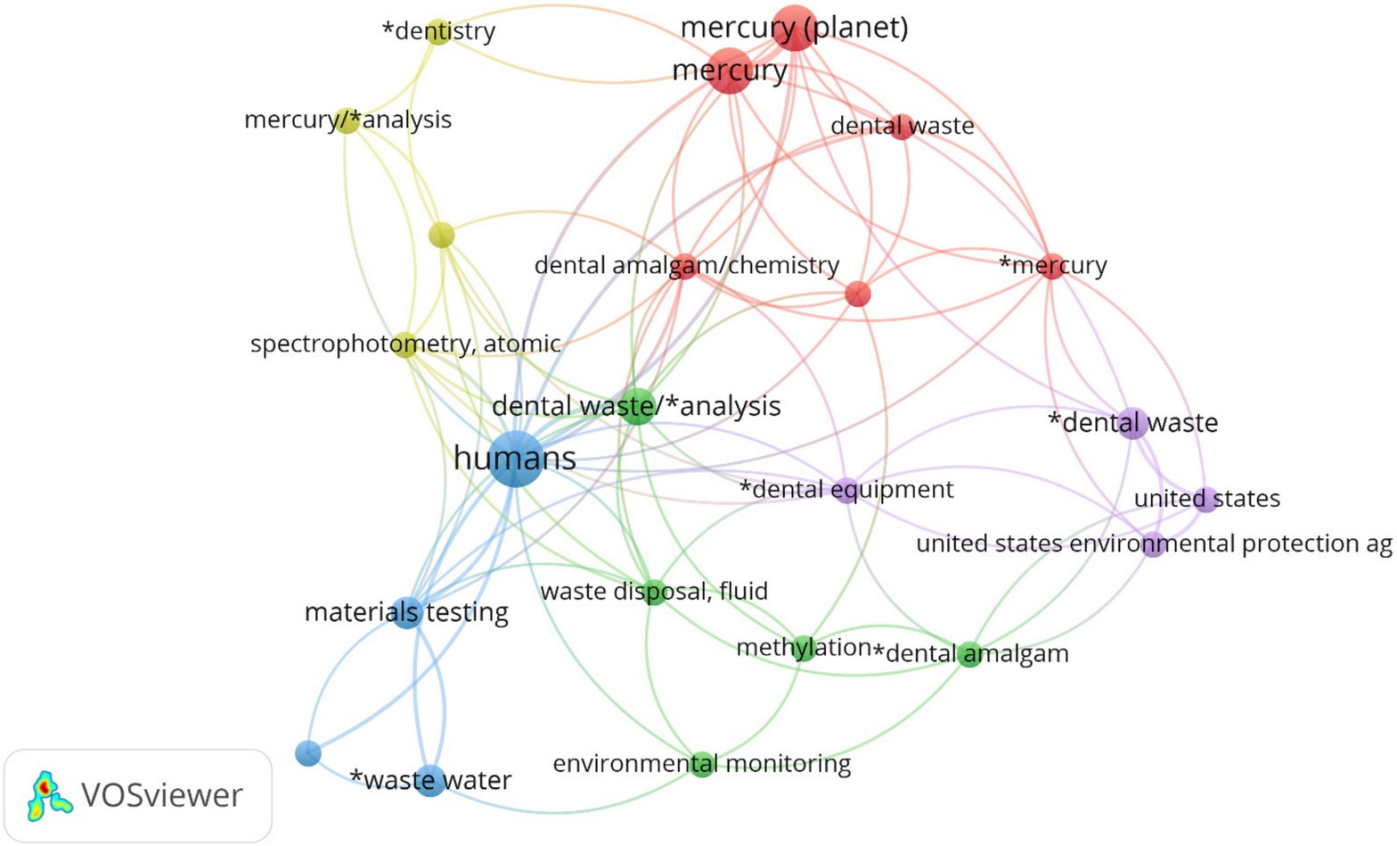
Keyword co‐occurrence network.

The term “Humans” was the most frequent (nine occurrences), followed by “mercury” (six occurrences) and “wastewater” (four occurrences) (Figure 3). A total of 137 eligible high‐frequency keywords were identified, where each circle represents three occurrences of a keyword. The lines indicate that at least one article mentioned both keywords, and their distance reflects the relationship between articles that share references and citations. It is observed that “amalgam” and “mercury” appear in all distances. The words were grouped into five clusters, each represented by a color, to compose a visualization matrix of the high‐frequency keywords and their references in the literature. The red cluster groups terms such as “mercury,” “dental waste,” and “dental amalgam/chemistry,” highlighting the relationship between Hg, dental waste, and the chemical and environmental implications of dental amalgam. The blue cluster, which includes “humans,” “wastewater,” and “materials testing,” focuses on the analysis of liquid dental waste and its environmental impacts. The green cluster, with words such as “environmental monitoring,” “waste disposal, fluid,” and “methylation*dental amalgam,” deals with environmental monitoring and proper disposal of Hg‐containing waste. The purple cluster, with terms such as “United States,” “United States Environmental Protection ag,” and “*dental equipment,” addresses environmental regulations and policies associated with Hg contamination in dentistry. Finally, the yellow cluster, with words such as “mercury/*analysis,” “spectrophotometry, atomic,” and “*dentistry,” is related to chemical analysis and laboratory techniques used to detect Hg in dental materials.

## 4 Discussion

This is the first study to map evidence about DWW, attempting to elucidate its risks without restricting the focus solely to Hg analyses. The included studies reveal that DWW contains a variety of toxic compounds, including heavy metals, residues from restorative materials, and organic substances with biological activity. In addition to Hg and methylmercury (MeHg), substances originating from resin‐based materials, such as residual monomers and BPA, were identified, indicating a broader risk profile than previously recognized.

None of the included studies categorizes DWW as nontoxic, and none of them conclude that there is no cause for concern in dental clinics or offices. Most studies highlight the toxic potential of DWW for the environment, even in the face of a lack of specific regulations in many countries. Compounds present in DWW are often discharged directly into the urban sewage system, which poses a threat to the integrity of water bodies and aquatic organisms. The risks include cytotoxic effects, bactericides and contaminants, and endocrine disruption, as demonstrated by studies (Cataldi et al. 2017; Rani et al. 2015; Mulligan et al. 2021; Shraim et al. 2011).

The waste and microparticles are removed through dental suction and discharged into the sewer, reinforcing the importance of containment strategies within the dental chair itself (Rani et al. 2015; Binner et al. 2022; Bristela et al. 2012; Rani et al. 2015).

This study aligns with global efforts toward sustainable practices regarding the environment. The United Nations' global development agenda until 2030, which includes the Sustainable Development Goals, prioritizes Goal 6, focusing on clean water and sanitation. This agenda encourages countries to adapt to ensure a more just, dignified, inclusive, and sustainable world (Kronemberger 2019). The increasing environmental awareness, or eco‐consciousness, observed in recent years reflects a global progressive movement toward mitigating environmental damage. This translates into the incorporation of sustainability concepts and environmentally friendly solutions in various areas of the economy and labor (Mulimani 2017).

Special attention was given to the included studies that investigated resin‐based or so‐called metal‐free materials (Binner et al. 2022; Mulligan et al. 2021; Polydorou et al. 2020; Reidelbach et al. 2021)^.^ These studies provide valuable insight into this pollution pathway, showing that monomer eluates—such as bisphenol A (BPA)—can be detected in DWW and released into the environment, with reported toxicological effects including bactericidal and cytotoxic activity in vitro (on bacteria and human cells) as well as estrogenic effects (Reidelbach et al. 2021). Clinically, polishing, replacing, or adjusting restorations generates particulate debris that is captured by the dental suction system and discharged to wastewater, potentially reaching the environment via municipal sewer systems (Mulligan et al. 2021).

Prevention techniques for toxic effluent have been identified in the studies included in this analysis. With the advancements in dental facilities, the filtration of amalgam waste can now be applied in many offices worldwide (Olivera et al. 2020). These separators or filters use a variety of techniques to trap amalgam residues chairside, reducing the amalgam composition in the effluent from DWW where Hg or MeHg can leach over time. Previous studies (Adegbembo et al. 2002; Albishri and Yakout 2023; Olivera et al. 2020; Pederson et al. 1999; Reed et al. 2002, 2004; Scarano et al. 2020; Stone et al. 2008) have assessed the effectiveness of these separators or filters in retaining amalgam residues. Furthermore, a recent study (Polydorou et al. 2020) stated that these separators are capable of filtering new materials, not limited to amalgam retention alone.

Furthermore, the feasibility and unique added value of on‐site treatment strategies (such as the separators discussed previously) address a critical gap that municipal WWTPs cannot. As highlighted by Cataldi et al. (2017), DWW is unsuitable for introduction into the urban sewage system as household waste. The core issue is that DWW represents a “point source” of pollution: a low volume of wastewater containing high concentrations of specific, complex contaminants (e.g., heavy metals, microplastics, and BPA). These pollutants are subsequently discharged into the municipal sewer system, where they are highly diluted. This dilution is a challenge, as conventional WWTPs, which are generally designed to treat biological waste, are not optimized to efficiently remove these specific contaminants. Studies show that WWTP removal efficiency for BPA can be highly variable and incomplete (Guerra et al. 2015; Taofiq et al. 2022), and even high removal rates for microplastics still result in millions of particles being released daily into aquatic environments (Magni et al. 2020). This inefficiency in handling diluted pollutants is precisely why source control rather than reliance on WWTPs is strongly recommended for dental effluents (Zhao et al. 2012). Consequently, dental pollutants tend to either “pass through” the treatment process and are released directly into receiving water bodies, or they accumulate in the sewage sludge (biosolids) (Magni et al. 2020). This accumulation in the sludge is particularly problematic, as this material is often applied to agricultural land as fertilizer, creating a pathway for toxic metals and microplastics to contaminate terrestrial environments and, potentially, the human food chain (Corradini et al. 2019; McBride 2003). Therefore, the implementation of on‐site treatment (e.g., amalgam separators, filters) is the most viable and effective strategy, as it captures contaminants while they are still concentrated, preventing their wider, untreatable dissemination.

The methodologies used in the studies analyzed varied widely, which made direct comparisons between the results difficult. It was observed that some studies collected dental effluent (DWW) in real clinical settings, while others used simulated laboratory scenarios. Despite these differences, all articles adequately described their testing and validation methods, following recognized guidelines. The most widely used method for assessing mercury concentration was cold vapor atomic absorption spectrophotometry. For the analysis of physical–chemical parameters, such as pH, conductivity, and turbidity, the studies followed the procedures established by the Standard Methods for the Examination of Water and Wastewater. The lack of standardization in the collection methods and measurement units limits the possibility of generalizing the data. However, several analytical techniques were used, including mass spectrometry and in vitro cytotoxicity tests, estrogenicity (Reidelbach et al. 2021), and genetic and metagenomic analyses (Jiao et al. 2023), which demonstrates an attempt to deepen scientific knowledge on the topic.

Most of the studies were conducted in the United States, where environmental regulatory agencies have progressively implemented mandatory requirements for the installation of amalgam separators in dental clinics, by ISO 11143 standards. These devices have proven effective in retaining Hg and, according to recent studies, may also filter other contaminants such as composite resin particles (Batchu et al. 2006). Such regulations aim to minimize the release of hazardous substances into the environment and have stimulated research and the development of innovative technologies in the sector. However, the analysis highlights a critical regulatory gap in many other regions, where the absence of clear and mandatory guidelines for the disposal of DWW may lead to inadequate practices, posing risks to both environmental and public health.

After establishing that DWW is toxic, it becomes essential to discuss regulations related to the treatment of this sewage by countries. Some research reports the challenges faced by national regulations in their respective countries (Cataldi et al. 2017). In the absence of specific laws, the management of these wastes can become flexible. This study highlights a critical gap in the regulation of wastewater from dental units worldwide. Cataldi et al. (2017) emphasize that effluents from dental units are unsuitable for introduction into the urban sewage system as household waste. This highlights the importance of adhering to national and international environmental regulations regarding these wastes.

Although this study focuses on the analysis of potential impacts on human health, the available literature on this topic remains limited. Few studies have investigated adverse health outcomes, such as elevated urinary mercury levels following the removal of amalgam restorations, or the detection of antimicrobial resistance genes (ARGs) and mobile genetic elements (MGEs) in DWW. Despite the paucity of evidence, these findings underscore the urgent need for more comprehensive and methodologically robust investigations aimed at evaluating both direct and indirect human exposure to contaminants present in DWW.

Although the title of this study refers to potential impacts on human health, the literature remains scarce in this regard. The available evidence is limited and insufficient to draw firm conclusions. Only a few studies have examined possible adverse effects, such as increased urinary total mercury (tHg) levels after removal of amalgam restorations (Oliveira et al. 2014), or the detection of ARGs and MGEs in DWW (Jiao et al. 2023). Despite the small number of studies, these findings underscore the urgent need for robust investigations assessing direct and indirect human exposure to contaminants present in DWW.

Some methodological limitations must be acknowledged. The search strategy, although based on clear and reproducible criteria, was restricted to three databases and to the English language, which may have limited the comprehensiveness of the review. We also did not include gray literature, regulatory documents, or nonindexed reports, which may have led to the omission of relevant evidence, particularly from low‐ and middle‐income countries. Furthermore, many initially identified articles were excluded for addressing dental unit waterlines rather than DWW itself. Although this decision increased the conceptual focus of the review, it may have excluded studies that indirectly reported outcomes related to DWW. Another major challenge was the methodological heterogeneity among the included studies, both in terms of study designs and the variables analyzed. This hindered direct comparisons among findings and limited the possibility of conducting more robust quantitative analyses. As this is a scoping review, we did not perform a formal risk of bias or quality appraisal of individual studies; therefore, our findings should be interpreted as a mapping of existing evidence rather than a graded estimate of effect or causality. In addition, the predominance of studies conducted in high‐income countries, often in academic or large clinical settings, may limit the generalizability of our synthesis to other regulatory and infrastructural contexts.

Finally, there is an urgent need for new studies that investigate the risks to human health associated with DWW exposure, particularly considering the presence of emerging contaminants and the growing concern regarding antimicrobial resistance. Future research should prioritize longitudinal and experimental designs, standardized sampling and analytical protocols, and explicit evaluation of exposure pathways and health outcomes, so that dose–response relationships and health‐based thresholds can be more accurately defined.

## 5 Conclusion

Based on the findings of this study, it becomes evident that DWW represents a significant environmental hazard due to its toxic components and polluting potential. The evidence indicates negative impacts on aquatic ecosystems, reinforcing the urgency of implementing regulatory and management strategies to prevent further environmental degradation and protect public health.

Given the limited number and heterogeneity of available studies, these conclusions should be interpreted with caution and as hypothesis‐generating rather than definitive evidence on human health impacts. Although the number of studies assessing human health risks remains limited, a critical review of the available data suggests possible concerns, such as exposure to heavy metals (e.g., mercury), BPA, and antimicrobial‐resistant microorganisms. These findings, albeit preliminary, signal a potential health threat and highlight crucial gaps in the literature.

Therefore, there is a pressing need for more robust and methodologically sound research to clarify exposure pathways, dose–response relationships, and long‐term health outcomes. Such evidence is crucial in supporting risk assessments and informing public health policies.

In line with the evidence mapped in this scoping review, future work on DWW should move forward in three complementary directions: development and evaluation of chairside or point‐of‐generation technologies to retain or treat pollutants before discharge; systematic monitoring and characterization of DWW, including emerging contaminants and antimicrobial resistance; and implementation of good‐practice protocols in dental services to reduce pollutant release at the source. Stating these priorities may help guide both researchers and regulators.

Ultimately, this study underscores the importance of a holistic and interdisciplinary approach to addressing the challenges posed by DWW. Integrating scientific research, environmental regulation, and clinical practice is crucial to achieving sustainable solutions and promoting a healthier environment for future generations.

## Author Contributions

**Giordana Picolo Furini:** conceptualization, writing – original draft, writing – review and editing. **Rafaela Munz Belarmino:** writing – review and editing. **Lilian Rigo:** supervision, project administration, funding acquisition.

## Funding

The authors have nothing to report.

## Ethics Statement

The authors have nothing to report.

## Consent

All authors mentioned in the manuscript have agreed to authorship and participation in the manuscript. All authors have read and agreed to the published version of the manuscript.

## Conflicts of Interest

The authors declare no conflicts of interest.

## Data Availability

The authors declare that the data supporting the findings of this study are available within the paper.
